# Supplementary material for: Unveiling the Bioactive Potential of the Invasive Jellyfish Phyllorhiza punctata Through Integrative Transcriptomic and Proteomic Analyses
Source: Biomolecules. 2025 Aug 4;15(8):1121. doi: 10.3390/biom15081121 (PMC12383608; doi:10.3390/biom15081121)
Supplement: Supplementary file 1 [file biomolecules-15-01121-s001.zip › Suplementary Material/FileS3/PEPFOLD4/XP_065060802_14099/clusters.html]

 PEP-FOLD clusters


Download archive of all models  here   

|  |  |  |
| --- | --- | --- |
| # | file | sOPEP |
| # cluster 1 - Size: 19 |  |  |
|  | XP\_065060802\_14099-model1 | -19.1724 |
|  | XP\_065060802\_14099-model1.1 | -18.9919 |
|  | XP\_065060802\_14099-model1.2 | -18.9652 |
|  | XP\_065060802\_14099-model1.3 | -18.9452 |
|  | XP\_065060802\_14099-model1.4 | -18.9452 |
|  | XP\_065060802\_14099-model1.5 | -18.9241 |
|  | XP\_065060802\_14099-model1.6 | -18.9174 |
|  | XP\_065060802\_14099-model1.7 | -18.8702 |
|  | XP\_065060802\_14099-model1.8 | -18.8427 |
|  | XP\_065060802\_14099-model1.9 | -18.7863 |
|  | XP\_065060802\_14099-model1.10 | -18.7579 |
|  | XP\_065060802\_14099-model1.11 | -18.7227 |
|  | XP\_065060802\_14099-model1.12 | -18.7018 |
|  | XP\_065060802\_14099-model1.13 | -18.6706 |
|  | XP\_065060802\_14099-model1.14 | -18.6682 |
|  | XP\_065060802\_14099-model1.15 | -18.5956 |
|  | XP\_065060802\_14099-model1.16 | -18.5711 |
|  | XP\_065060802\_14099-model1.17 | -18.3352 |
|  | XP\_065060802\_14099-model1.18 | -17.3647 |
| # cluster 2 - Size: 27 |  |  |
|  | XP\_065060802\_14099-model2 | -15.0927 |
|  | XP\_065060802\_14099-model2.1 | -14.6627 |
|  | XP\_065060802\_14099-model2.2 | -14.6429 |
|  | XP\_065060802\_14099-model2.3 | -14.5973 |
|  | XP\_065060802\_14099-model2.4 | -14.5157 |
|  | XP\_065060802\_14099-model2.5 | -14.5157 |
|  | XP\_065060802\_14099-model2.6 | -14.5157 |
|  | XP\_065060802\_14099-model2.7 | -14.5157 |
|  | XP\_065060802\_14099-model2.8 | -14.5157 |
|  | XP\_065060802\_14099-model2.9 | -14.5157 |
|  | XP\_065060802\_14099-model2.10 | -14.5157 |
|  | XP\_065060802\_14099-model2.11 | -14.4694 |
|  | XP\_065060802\_14099-model2.12 | -14.4694 |
|  | XP\_065060802\_14099-model2.13 | -14.3854 |
|  | XP\_065060802\_14099-model2.14 | -14.3827 |
|  | XP\_065060802\_14099-model2.15 | -14.3827 |
|  | XP\_065060802\_14099-model2.16 | -14.3524 |
|  | XP\_065060802\_14099-model2.17 | -14.3524 |
|  | XP\_065060802\_14099-model2.18 | -14.3292 |
|  | XP\_065060802\_14099-model2.19 | -14.213 |
|  | XP\_065060802\_14099-model2.20 | -14.0156 |
|  | XP\_065060802\_14099-model2.21 | -13.8327 |
|  | XP\_065060802\_14099-model2.22 | -10.7812 |
|  | XP\_065060802\_14099-model2.23 | -9.61861 |
|  | XP\_065060802\_14099-model2.24 | -8.28961 |
|  | XP\_065060802\_14099-model2.25 | -7.49997 |
|  | XP\_065060802\_14099-model2.26 | -6.24441 |
| # cluster 3 - Size: 2 |  |  |
|  | XP\_065060802\_14099-model3 | -10.2335 |
|  | XP\_065060802\_14099-model3.1 | -6.09525 |
| # cluster 4 - Size: 5 |  |  |
|  | XP\_065060802\_14099-model4 | -7.18329 |
|  | XP\_065060802\_14099-model4.1 | -6.84649 |
|  | XP\_065060802\_14099-model4.2 | -6.38148 |
|  | XP\_065060802\_14099-model4.3 | -6.11584 |
|  | XP\_065060802\_14099-model4.4 | -5.25674 |
| # cluster 5 - Size: 9 |  |  |
|  | XP\_065060802\_14099-model5 | -6.89756 |
|  | XP\_065060802\_14099-model5.1 | -5.51539 |
|  | XP\_065060802\_14099-model5.2 | -5.32672 |
|  | XP\_065060802\_14099-model5.3 | -5.26915 |
|  | XP\_065060802\_14099-model5.4 | -5.22269 |
|  | XP\_065060802\_14099-model5.5 | -5.18352 |
|  | XP\_065060802\_14099-model5.6 | -5.14321 |
|  | XP\_065060802\_14099-model5.7 | -5.10559 |
|  | XP\_065060802\_14099-model5.8 | -5.10475 |
| # cluster 6 - Size: 2 |  |  |
|  | XP\_065060802\_14099-model6 | -6.73869 |
|  | XP\_065060802\_14099-model6.1 | -6.68179 |
| # cluster 7 - Size: 3 |  |  |
|  | XP\_065060802\_14099-model7 | -6.34392 |
|  | XP\_065060802\_14099-model7.1 | -6.0154 |
|  | XP\_065060802\_14099-model7.2 | -5.76396 |
| # cluster 8 - Size: 3 |  |  |
|  | XP\_065060802\_14099-model8 | -6.32218 |
|  | XP\_065060802\_14099-model8.1 | -6.0297 |
|  | XP\_065060802\_14099-model8.2 | -5.46671 |
| # cluster 9 - Size: 1 |  |  |
|  | XP\_065060802\_14099-model9 | -6.1914 |
| # cluster 10 - Size: 3 |  |  |
|  | XP\_065060802\_14099-model10 | -6.13247 |
|  | XP\_065060802\_14099-model10.1 | -5.84602 |
|  | XP\_065060802\_14099-model10.2 | -5.582 |
| # cluster 11 - Size: 3 |  |  |
|  | XP\_065060802\_14099-model11 | -6.01115 |
|  | XP\_065060802\_14099-model11.1 | -5.89457 |
|  | XP\_065060802\_14099-model11.2 | -5.65237 |
| # cluster 12 - Size: 1 |  |  |
|  | XP\_065060802\_14099-model12 | -5.91718 |
| # cluster 13 - Size: 1 |  |  |
|  | XP\_065060802\_14099-model13 | -5.91468 |
| # cluster 14 - Size: 2 |  |  |
|  | XP\_065060802\_14099-model14 | -5.80809 |
|  | XP\_065060802\_14099-model14.1 | -5.7391 |
| # cluster 15 - Size: 7 |  |  |
|  | XP\_065060802\_14099-model15 | -5.66068 |
|  | XP\_065060802\_14099-model15.1 | -5.34055 |
|  | XP\_065060802\_14099-model15.2 | -5.29879 |
|  | XP\_065060802\_14099-model15.3 | -5.23652 |
|  | XP\_065060802\_14099-model15.4 | -5.22339 |
|  | XP\_065060802\_14099-model15.5 | -5.21475 |
|  | XP\_065060802\_14099-model15.6 | -5.20512 |
| # cluster 16 - Size: 2 |  |  |
|  | XP\_065060802\_14099-model16 | -5.46948 |
|  | XP\_065060802\_14099-model16.1 | -5.25019 |
| # cluster 17 - Size: 1 |  |  |
|  | XP\_065060802\_14099-model17 | -5.40033 |
| # cluster 18 - Size: 3 |  |  |
|  | XP\_065060802\_14099-model18 | -5.35941 |
|  | XP\_065060802\_14099-model18.1 | -5.33066 |
|  | XP\_065060802\_14099-model18.2 | -5.23489 |
| # cluster 19 - Size: 1 |  |  |
|  | XP\_065060802\_14099-model19 | -5.35897 |
| # cluster 20 - Size: 1 |  |  |
|  | XP\_065060802\_14099-model20 | -5.34638 |
| # cluster 21 - Size: 1 |  |  |
|  | XP\_065060802\_14099-model21 | -5.28359 |
| # cluster 22 - Size: 2 |  |  |
|  | XP\_065060802\_14099-model22 | -5.2814 |
|  | XP\_065060802\_14099-model22.1 | -5.15075 |
| # cluster 23 - Size: 1 |  |  |
|  | XP\_065060802\_14099-model23 | -5.19077 |
|  |  |  |
